# The Effect of Badger Culling on Breakdown Prolongation and Recurrence of Bovine Tuberculosis in Cattle Herds in Great Britain

**DOI:** 10.1371/journal.pone.0051342

**Published:** 2012-12-07

**Authors:** Katerina Karolemeas, Christl A. Donnelly, Andrew J. K. Conlan, Andrew P. Mitchell, Richard S. Clifton-Hadley, Paul Upton, James L. N. Wood, Trevelyan J. McKinley

**Affiliations:** 1 Disease Dynamics Unit, Department of Veterinary Medicine. University of Cambridge, Cambridge, United Kingdom; 2 MRC Centre for Outbreak Analysis and Modelling, Department of Infectious Disease Epidemiology, Imperial College, London, United Kingdom; 3 Animal Health and Veterinary Laboratories Agency, Weybridge, Surrey, United Kingdom; Hopital Raymond Poincare - Universite Versailles St. Quentin, France

## Abstract

Bovine tuberculosis is endemic in cattle herds in Great Britain, with a substantial economic impact. A reservoir of *Mycobacterium bovis* within the Eurasian badger (*Meles meles*) population is thought to have hindered disease control. Cattle herd incidents, termed breakdowns, that are either ‘*prolonged*’ (lasting ≥240 days) or ‘*recurrent*’ (with another breakdown within a specified time period) may be important foci for onward spread of infection. They drain veterinary resources and can be demoralising for farmers. Randomised Badger Culling Trial (RBCT) data were re-analysed to examine the effects of two culling strategies on breakdown prolongation and recurrence, *during* and *after* culling, using a Bayesian hierarchical model. Separate effect estimates were obtained for the ‘core’ trial areas (where culling occurred) and the ‘buffer’ zones (up to 2 km outside of the core areas). For breakdowns that started during the culling period, ‘*reactive*’ (localised) culling was associated with marginally increased odds of prolongation, with an odds ratio (OR) of 1.7 (95% credible interval [CI] 1.1–2.4) within the core areas. This effect was not present after the culling ceased. There was no notable effect of ‘*proactive*’ culling on prolongation. In contrast, reactive culling had no effect on breakdown recurrence, though there was evidence of a reduced risk of recurrence in proactive core areas during the culling period (ORs and 95% CIs: 0.82 (0.64–1.0) and 0.69 (0.54–0.86) for 24- and 36-month recurrence respectively). Again these effects were not present after the culling ceased. There seemed to be no effect of culling on breakdown prolongation or recurrence in the buffer zones. These results suggest that the RBCT badger culling strategies are unlikely to reduce either the prolongation or recurrence of breakdowns in the long term, and that reactive strategies (such as employed during the RBCT) are, if anything, likely to impact detrimentally on breakdown persistence.

## Introduction

Bovine tuberculosis (bTB), caused by *Mycobacterium bovis*, is endemic in Great Britain (GB). A routine surveillance programme to slaughter cattle classified as infected has been unsuccessful, with incidence of herd ‘breakdowns’ (movement restrictions associated with detection of infection in cattle) increasing over the last 25 years [1]. Failure to eradicate bTB from GB has been complicated by the existence of a wildlife reservoir, namely the Eurasian badger (*Meles meles*) [2]–[4].

Nationally, around 30% of herd breakdowns are ‘*prolonged*’ (≥240 days) [5], and around 23% and 38% are ‘*recurrent*’ within 12 and 24 months respectively [6]. These persistent breakdowns are important as they are demanding on resources, and may additionally be acting as foci of infection, fuelling the increase in incidence. Furthermore, they can have a substantially detrimental effect on the well-being of farmers [7]. Breakdowns may become persistent from the presence of underlying and undetected infection within the herd, or by transmission and re-infection into the herd from other herds or environmental reservoirs of infection. The relative contribution of the badger reservoir to these empirical measures of persistence is not clear.

Due to a lack of detailed data on badger densities and infection status (which is not routinely collected), many previous studies have focussed on measuring associations between proxies for badger risk and incidence of bTB. For example, in the Republic of Ireland (ROI) [8] there was found to be an association between the presence of badgers on farms and breakdowns that were either over 12 months in duration, or recurrent within a four-year period. In another study [9] there was found to be no association with the presence of badgers and recurrence at the test conducted six months after the end of a breakdown. In GB, an association between the relative density of badgers and breakdowns over six months duration has been reported [10]. Although the aforementioned studies were similar in that they all used bTB test-negative herds as controls, differences in their case definitions for persistence makes comparisons between the findings challenging.

Previous studies in GB that examined risk factors for breakdown prolongation and recurrence, in which a range of farm-level factors were considered, included examination of information on the presence or absence of badgers, and whether or not badger control policies were performed at the farm level [5], [6]. Although no association was found between the badger variables examined and breakdown persistence, it is possible that this lack of association may have been due to confounding in measured or unmeasured variables, or that some of the variables identified in the model represented proxies for increased potential for transmission from badgers.

Further insight into the role that badgers play in infecting cattle herds can be gained from examining the effect of badger culling. Since 1971, when a dead badger infected with *M. bovis* was first discovered on a farm affected by bTB in GB, badgers have been strongly implicated in the transmission of *M. bovis* to cattle, prompting a number of badger culling strategies that occurred between 1973 and 1998. However, these culling operations [11], and those conducted in the ROI [12], [13] lacked randomised control areas where no culling was conducted, making conclusions difficult to interpret.

The Randomised Badger Culling Trial (RBCT) was set up in 1998 to examine the effect of badger culling on bTB incidence in cattle herds in GB, and specifically included randomly selected matched control areas where no culling was undertaken [14]. The RBCT was designed and conducted by the Independent Scientific Group on Cattle TB (ISG; [14]). The data are a valuable resource, and various analyses have been conducted. Analyses to date have measured the effect of culling on *overall confirmed* and *total* (confirmed and unconfirmed) *incidence*, both during and subsequent to the trial [15]–[17], and more recently have examined individual herd risk factors for breakdowns [18], [19]. Nonetheless, the effect of badger culling on *persistent* breakdowns within individual cattle herds has yet to be examined.

Widespread badger culling remains illegal in GB and is an ongoing subject of political debate [4], [14], [20]. However, in December 2011 Defra ministers announced a cull of badgers in two pilot areas, originally due to commence in 2012 [21], but now delayed until 2013 [22]. Farmers and landowners in these areas will be able to apply for licences to reduce badger populations at their own expense, and the humaneness of the culling will be judged by a panel of independent experts at the end of the period. The results from the pilot areas will inform policy decisions on whether this approach will be more widely adopted in the future. As an alternative, the vaccination of badgers is currently being trialled in one area of Gloucester [23], which is planned to continue until 2015, and in June 2012 the Welsh Assembly Government announced that a badger vaccination trial had begun as part of their bTB eradication strategy [24].

The perceived failure to address the wildlife reservoir has led to much distress and unrest in farming communities. Farmers are often reluctant to implement increased cattle controls when re-infection by badgers is perceived to be inevitable. Knowledge of the role of badgers in the re-infection of cattle herds is critical to inform those developing control policies. In this study we quantify the effects of the two badger culling strategies (proactive and reactive) conducted during the RBCT, on breakdown prolongation and recurrence in individual herds in areas of high cattle bTB incidence in GB.

**Table 1:** Numbers of cases (prolonged) and controls (non-prolonged), and the proportion prolonged, for the different treatment areas aggregated across the core and buffer zones.

	Core				Buffer			
Treatment	Cases	Controls	Total	Proportion	Cases	Controls	Total	Proportion
Proactive	460	893	1353	0.34	371	650	1021	0.36
Reactive	437	750	1187	0.37	298	535	833	0.36
Survey	686	1214	1900	0.36	399	796	1195	0.33

## Materials and Methods

### Summary of RBCT Trial Areas and Culling Treatments

The RBCT was conducted in 30 trial areas, located in areas of high bTB incidence, mainly in the West and South-west of England [14], [15]. Trial areas were grouped into triplets of three core areas (each approximately 100 km^2^), surrounded by buffers to ensure that the trial area boundaries were at least 3 km apart [14], [15]. Within each triplet, each core area received one of three treatments.

**Table 2:** Odds Ratios and 95% credible intervals– relative to a baseline of survey-only core areas–for prolongation in the different treatment areas in the periods during and after the culling; adjusted for breakdown confirmation status.

	Core		Buffer	
	During	After	During	After
**Survey**			0.95 (0.76–1.1)	
**Reactive**	1.7 (1.1–2.4)	0.97 (0.79–1.2)	1.2 (0.69–2.1)	1.2 (0.92–1.5)
**Proactive**	1.1 (0.87–1.4)	0.98 (0.79–1.2)	1.3 (0.94–1.7)	1.0 (0.79–1.4)

Results are further stratified into core and buffer zones.

*Proactive* culling was conducted across all accessible land with the aim of using annual culling to reduce badger density to the greatest extent possible within the constraints of welfare and logistical considerations. The first proactive culls occurred between 1998 and 2002 (depending on the triplet) and culling was repeated approximately once yearly (the total number of culls ranged from 4 to 7 across the ten triplets) to maintain the badger population at as low a level as possible. The last proactive cull in each triplet was in 2005.

**Table 3:** Numbers of cases (recurrent) and controls (non-recurrent), and the proportion recurrent, for the different treatment areas aggregated across the core and buffer zones and stratified by follow-up.

		Core				Buffer			
Follow-up	Treatment	Cases	Controls	Total	Proportion	Cases	Controls	Total	Proportion
	Proactive	309	676	985	0.31	209	505	714	0.29
12 months	Reactive	338	616	954	0.35	251	427	678	0.37
	Survey	479	900	1379	0.35	266	571	837	0.32
	Proactive	458	513	971	0.47	351	373	724	0.48
24 months	Reactive	498	442	940	0.53	336	316	652	0.52
	Survey	698	697	1395	0.50	436	439	875	0.50
	Proactive	475	360	835	0.57	370	262	632	0.59
36 months	Reactive	524	283	807	0.65	342	210	552	0.62
	Survey	768	477	1245	0.62	458	328	786	0.58

*Reactive* culling was conducted in response to a confirmed breakdown (evidence of visible bTB lesions post-mortem or *M. bovis* cultured in at least one slaughtered animal) with the aim to remove all badger social groups in a localised area that might have access to the breakdown farm. The first reactive cull occurred between 1999 and 2003 (depending on the triplet). Reactive culling was suspended in November 2003 due to evidence of increased incidence of bTB in cattle herds in the these areas observed at a planned interim analysis [15].

In *survey-only* areas badger activity was documented but no culling was conducted as part of the trial. These areas acted as control areas for the proactive and reactive areas. For both proactive and reactive culling treatments badgers were caught in cage traps and killed by gunshot [14].

**Table 4:** Odds Ratios and 95% credible intervals (in parentheses)–relative to a baseline of survey-only core areas–for recurrence in the different treatment areas in the periods during and after the culling; adjusted for herd size, number of reactors, and breakdown history in the previous three years.

		Core		Buffer	
Follow-up	Treatment	During	After	During	After
	Survey			0.89 (0.71–1.1)	
12 months	Reactive	1.1 (0.73–1.6)	0.92 (0.74–1.1)	1.3 (0.77–2.0)	1.2 (0.93–1.6)
	Proactive	0.86 (0.66–1.1)	0.85 (0.66–1.1)	0.85 (0.60–1.2)	0.89 (0.65–1.2)
	Survey			1.0 (0.84–1.2)	
24 months	Reactive	1.1 (0.76–1.5)	1 (0.84–1.3)	0.86 (0.54–1.3)	1.1 (0.85–1.4)
	Proactive	0.82 (0.64–1.0)	0.99 (0.77–1.3)	0.88 (0.67–1.1)	0.96 (0.72–1.3)
	Survey			0.89 (0.72–1.1)	
36 months	Reactive	0.99 (0.70–1.4)	1.1 (0.86–1.4)	0.78 (0.48–1.2)	1.3 (0.99–1.7)
	Proactive	0.69 (0.54–0.86)	1.2 (0.84–1.5)	1.0 (0.75–1.3)	1.0 (0.72–1.5)

Models are fitted to each follow-up period (12, 24 and 36 months) separately. Results are further stratified into core and buffer zones.

### Data and Study Design

Data recorded in VetNet (the national GB surveillance database for bTB) were provided by the Animal Health and Veterinary Laboratories Agency, and consisted of all *breakdowns* that occurred in herds located within the RBCT core and buffer areas for the periods prior to, during and subsequent to the RBCT. In addition, we also obtained all the VetNet testing data for these herds over the same period (the last recorded test for one of these herds was 23^rd^ September 2011).

**Table 5:** Odds Ratios (ORs) and 95% credible intervals (in parentheses) of recurrence for the nuisance variables in the recurrent breakdown analyses.

Follow-up	Breakdown history	Max. herd size	Total no. of reactors
12 months	1.5 (1.3–1.7)	1 (0.97–1.1)	1.1 (1.1–1.2)
24 months	1.4 (1.2–1.6)	1 (0.93–1.1)	1.1 (1.1–1.2)
36 months	1.5 (1.3–1.8)	1 (0.97–1.1)	1.1 (1.1–1.2)

The OR for breakdown history is defined relative to having no breakdowns in the previous three years; the OR for the maximum herd size is per unit log-increase in herd size, and likewise for the total number of reactors. Models are fitted to each follow-up period (12, 24 and 36 months) separately.

For proactively and reactively culled treatment areas, breakdowns were eligible for inclusion if they started after the end of the first proactive or reactive cull, respectively, in each triplet. For the survey-only areas, breakdowns were eligible for inclusion in the study if they started after the end of the first proactive cull in the corresponding triplet. Full details of the timings of these events have been previously published [2], [25]. Also, only herds in the proactive and survey-only groups were included for Triplet J, since no reactive culling took place in this triplet.

**Figure 1:**
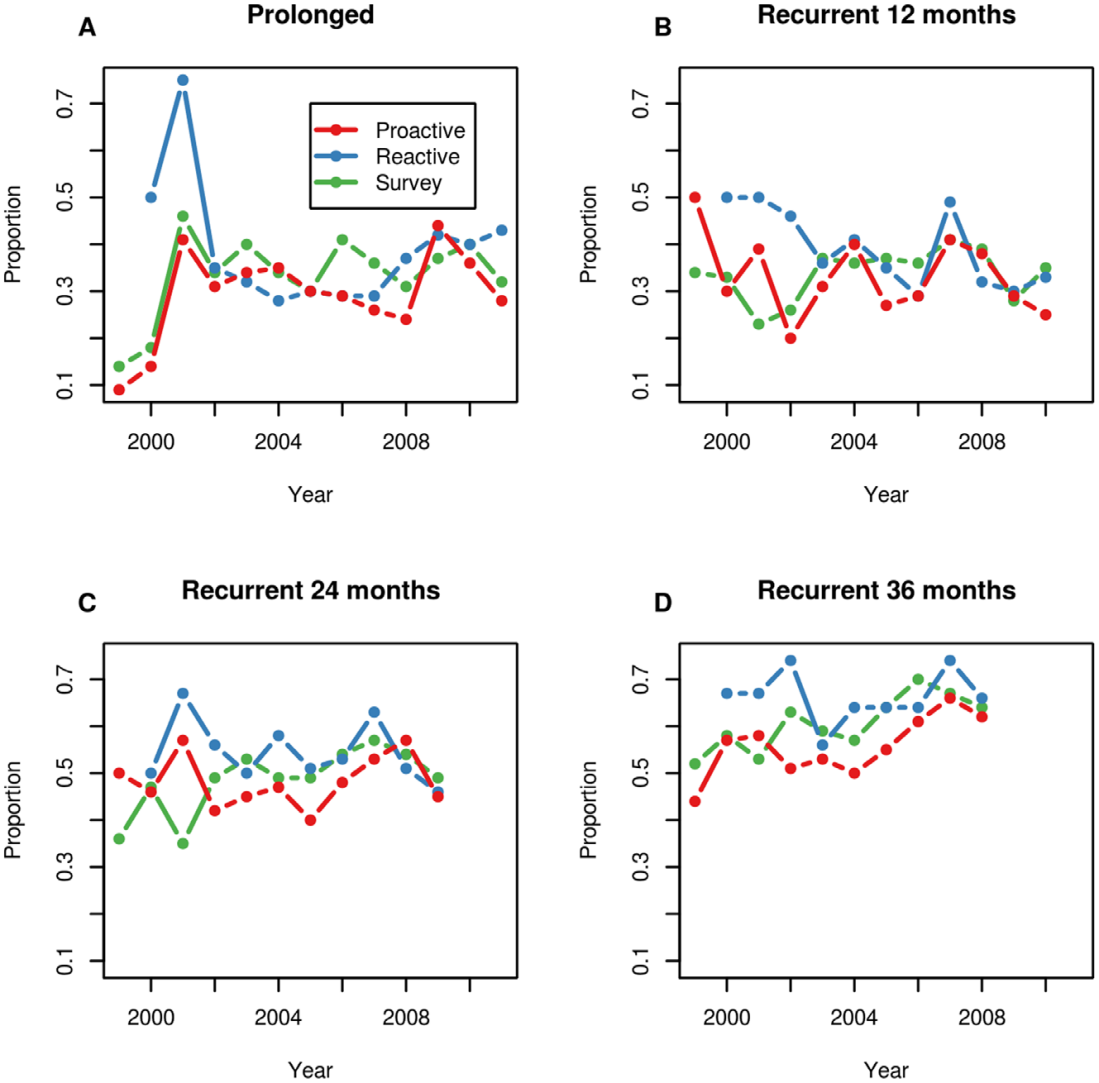
Plot showing the proportions of breakdowns starting in each year for each persistence category, stratified by culling treatment. Panels show prolonged breakdowns (**A**), recurrent breakdowns at 12 months (**B**), recurrent breakdowns at 24 months (**C**) and recurrent breakdowns at 36 months (**D**).

To examine the effect of badger culling on breakdown *prolongation*, breakdowns of duration ≥240 days were classified as cases (‘prolonged’) and those <240 days as controls (‘non-prolonged’), as justified in a previous study [5]. Where no end date was recorded in the data (indicative of an ongoing breakdown), breakdowns were excluded if the breakdown began <240 days before the last available test date (specified above), and classified as prolonged if it started at least 240 days before this date.

For the *recurrence* analysis, each breakdown was followed prospectively from its end date and classified as a case (‘recurrent’) if the herd experienced a further breakdown within a specified follow-up period (12, 24 or 36 months). Alternatively, a herd was classified as a control (‘non-recurrent’) if the herd experienced at least one herd-level test but did not suffer a further breakdown within the follow-up period. Breakdowns with insufficient follow-up (e.g. such as those that ended within 12 months of the last recorded test date for the 12 month analysis) were excluded. Full discussion of these definitions can be found in a previous study [6].

### Statistical Methods

The effect of proactive and reactive culling on breakdown prolongation and recurrence was evaluated using logistic regression models. Although all triplets were located in areas of high cattle bTB incidence, a triplet-level effect was included to account for potential between-triplet heterogeneity. In addition, since it was possible that individual herds could be included in the dataset more than once (if they had multiple breakdowns during the time period examined), an individual herd-level effect was incorporated to account for potential herd-level correlation, such that:
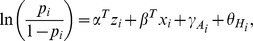
where 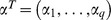 is a vector of regression parameters relating to a set of nuisance variables 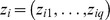 for breakdown *i*. Similarly, 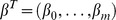 is a vector of regression parameters corresponding to a set of trial-specific variables 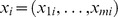. A triplet-level effect is represented by 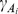, where 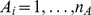 corresponds to the triplet containing breakdown *i*; and 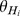 represents a herd-level effect where 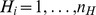 corresponds to the herd containing breakdown *i*.

The nuisance variables, 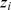, varied between the prolonged and recurrent analyses, and were chosen based on results from previous papers [5], [6]. For prolongation, the estimates were adjusted for the confirmation status of the breakdown, which had previously been identified to be by far the strongest variable associated with this measure of persistence [5]. By contrast, a combination of variables were identified as being associated with breakdown recurrence [6], and thus for the recurrence analyses, the estimates were adjusted for herd size (maximum herd size during the breakdown), recent breakdown history (a binary variable, taking the value 1 if the herd had experienced a breakdown in the previous three years, and 0 otherwise), and the total number of reactors during the breakdown. In order to linearise the relationship between the non-categorical confounding variables (herd size and total number of reactors) and the response variable, a log transformation was performed. To account for zeros in the data, and thus minimize bias in the covariates, 0.5 was added prior to the log transformation [26].

In each case the trial-specific variables were 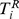, 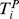, 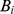 and 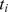, where 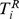 is a binary variable taking the value 1 if breakdown *i* was in a *reactive* area, and 0 otherwise; 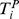 is likewise for *proactive* areas; 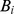 is a binary variable taking the value 1 if breakdown *i* is located in a *buffer* zone, and 0 if it is located in one of the core areas, and 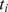 is a binary variable taking the value 1 if the breakdown started in the period *after* the cull, and 0 if it began *during* the cull. (Note here that 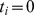 always in the survey-only areas, since no culling occurred.).

Therefore, the trial-specific component of the model is:
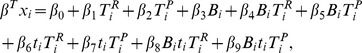
and the marginal log-odds ratios for the different comparisons were extracted through examining different combinations of the 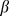 parameters.

The binary time variable was included in the model to adjust for the fact that the effect of culling on breakdown prolongation and recurrence may have differed in the periods *during* and *after* the culling treatments. The cut-offs for these classifications were derived from the known end dates of the cull in each triplet [25]. The interaction effects were included to assess the impact of each type of culling in the core and buffers zones during each of the two time periods.

The model was fitted to the data in a Bayesian framework using Markov chain Monte Carlo. The 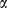 and 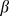 parameters were given vague 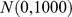 prior distributions (for the prolonged breakdown analysis) and 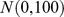 prior distributions (for the recurrent breakdown analysis). The triplet-level effects, 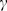, were given 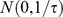 prior distributions with precision 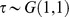, and the herd-level effects, 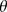, were given 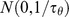 prior distributions and precision 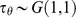. This Bayesian hierarchical framework is analogous to a random intercepts model in a frequentist framework (though we avoid the use of this terminology since in the Bayesian framework all parameters are considered to be random variables).

In each case a burn-in of 5,000 iterations was used, followed by 20,000 updates and the posterior distributions were thinned to return 1000 samples. Convergence was assessed by running multiple chains from different starting values (from overdispersed initial values) and examining the trace plots. In addition to this visual assessment, we checked that the Gelman-Rubin statistic 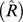 values were close to 1.0 [27].

Results are presented as odds ratios (ORs), with posterior means and 95% credible intervals (CI) reported to 2 significant figures. All analyses were carried out using the open-source R statistical package [28], except the fitting of the Bayesian model which was conducted in WinBUGS [29] using the R2WinBUGS package [30].

### 

## Results

Full model results for the 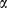 and 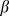 parameters are provided in Table S1.

### Breakdown Prolongation

A total of 7489 breakdowns were analysed in the model, comprising of 4440 in the core areas and 3049 in the buffer zones. The proportion of breakdowns that were prolonged was similar between each of the treatment areas in both the core and buffer zones (Table 1).

The marginal posterior mean OR and 95% CI for the impact of confirmation status on breakdown prolongation–obtained from the model fit–is 9.4 (7.9–11); consistent with previous results [5]. The marginal posterior mean ORs for the treatment effects (adjusted for the confirmation status of the breakdown) are shown in Table 2. There is some evidence of an increase in the odds of prolongation in the core reactive areas in the period during the cull (OR: 1.7; 95% C.I. [1.1–2.4]), though the effect size is slight (especially when compared to the effect of confirmation status), and it disappears after the culling period ends. There is no notable impact of either of the treatments on prolongation in the buffer zones.

### Breakdown Recurrence

A summary of the number of breakdowns included in each of the recurrence analyses (i.e. at 12, 24 and 36 months) is shown in Table 3. The overall sample size is similar for each of the three analyses, constituting 3318, 3306 and 2887 breakdowns in the core areas, for 12, 24 and 36 months respectively; and likewise 2229, 2251 and 1970 breakdowns in the buffer zones. However, the overall proportions of recurrent breakdowns increases as the follow-up period increases, with similar increases observed in each of the treatment areas for each follow-up period (Table 3).

Table 4 provides the marginal posterior mean ORs and 95% CIs for the treatment effects (adjusted for herd size, number of reactors, and breakdown history in the previous three years). There is no notable impact of culling treatment in the buffer zones, but there is a decrease in the odds of recurrence in the proactively culled core areas during the culling period, for both the 24 month (OR: 0.82; 95% CI [0.64–1.0]), and 36 month (OR: 0.69; 95% CI [0.54–0.86]) follow-up periods.

The marginal posterior mean ORs and 95% CIs for the adjusted variables are shown in Table 5 and herds that have experienced a breakdown in the previous three years, as well as those that have a larger number of reactors during the breakdown, are at increased risk of recurrence, consistent with previous findings [6]. Herd size is deemed less important here, which contrasts against other studies looking at different definitions of recurrence [31]–[33], but is consistent with previous results using the same definitions [6].

## Discussion

In this study we have quantified the effect of two badger culling strategies (proactive and reactive) on breakdown prolongation and recurrence in individual cattle herds in both the core areas and in the adjoining (and un-culled) buffer zones. We also explored the impacts of the culling treatment on persistence in two time periods: during the cull and after the cull.

In terms of breakdown prolongation, we found marginal evidence of an increase in the odds of prolongation in the core reactive areas in the period during the cull (OR: 1.7; 95% C.I. [1.1–2.4]). However, this detrimental effect did not persist in the period after the cull. There was no notable impact of culling treatment on prolongation in the buffer zones.

The mechanisms underlying these results are unclear. Both reactive and proactive culling have been shown to result in an increased prevalence of bTB infection in badger populations [34], most likely due to social and territorial disruption in mixing patterns in the badger populations as a result of the cull [35] and potentially leading to an increase in mixing and transmission between badgers and cattle [36], [37]. It is possible that these behaviours could result in an increase in the force-of-infection acting on an already infected cattle herd, and hence potentially increase the degree of within-herd spread of the disease.

In proactively culled areas, any increase in prevalence in badgers was likely to have been offset by a large reduction in badger density, which was not observed to anywhere near the same magnitude in the reactively culled areas [38]. Coupled with the fact that reactive culling was conducted over small, localised areas surrounding *confirmed* breakdowns, this may have resulted in increased contact between cattle and badgers in localised regions (i.e. herds) that were already experiencing above-average levels of underlying infection. This mechanism would also be consistent with the effect disappearing in the period after the culling ended, since there is evidence to suggest that re-colonisation (and hence stabilisation) of the badger populations was quick in the reactive areas [35], [37].

The perturbation effect hypothesis has been questioned by some who suggest a lag period is necessary before any effect might be expected to be seen [39]. The Godfray report [40], published in 2004, concluded that there was insufficient information in the reactive areas to support the perturbation effect hypothesis, questioning whether firm conclusions can be drawn from the reactively-culled areas due to the low numbers of badgers removed from relatively small areas. A similar consideration is whether reactive culling, on the scale conducted in the RBCT, would be able to influence breakdown prolongation and recurrence as measured in our study. However, subsequent analysis of cattle TB in and around proactively culled RBCT areas found a 29% increase in bTB risk observed among cattle herds living close to (but outside) proactive trial areas [17], consistent with the earlier finding in reactive areas. Our findings, albeit marginal, suggest that reactive culling, as practiced in the RBCT, is associated with a detrimental effect in the shorter term. This complements the results of a recent study [25], which examined the change in bTB risk in nearby cattle herds as a direct result of reactive culling over different time periods up to January 2007, concluding that the risk of having a confirmed breakdown was increased in the period during the reactive cull, even after adjusting for other important local risk factors.

Disruption to testing caused by the 2001 foot-and-mouth disease (FMD) epidemic is likely to have a more pronounced impact on the data obtained from reactive areas due to the timing of the culling periods with respect to the FMD outbreak. Reactive culling was stopped at a much earlier stage than the proactive cull, with all reactive areas experiencing their last cull in 2003, compared to 2005 for the proactive culls. In the model, the definition of the time period during the cull is centred around these earlier years in the reactive areas, which span the FMD epidemic. Figure 1A shows the proportions of prolonged breakdowns that started in each year, stratified by treatment. It can be seen that there was a spike in the levels of prolongation in each of the treatment regions in 2001, however the proportions of prolonged breakdowns were already higher in 2000 in the reactive areas compared to the proactive and survey-only areas, which carried through to higher levels in 2001. It is these patterns that are reflected in the increased OR for prolongation in the reactive areas during the culling period. However, there is no clear systematic or mechanistic reason why the reactive areas should have been disproportionately affected by FMD compared to the other areas. It is also worth noting that the sample sizes at these earlier time points is much smaller than at later time points (Table S2), and it is possible that the observed effect may simply be due to an artefact of the small sample size. However, we note that this should also be reflected in the uncertainty in the parameter estimates.

In terms of breakdown recurrence, we found a small decrease in the odds of recurrence at 24 and 36 months (ORs and 96% C.I.s of 0.82 [0.64–1.0] and 0.69 [0.54–0.86] respectively) in the proactively culled core areas in the period during the culling. These beneficial effects consequently reduced in the period subsequent to the cull. No notable impact of culling on recurrence was observed in the buffer zones. Recent work on the mechanisms of recurrence [41] suggest that due to the testing regime imposed upon a herd as a result of a breakdown, it is likely that re-introduction of infection (rather than persistence of infection within a herd) is the main driver of recurrence. To this end a large reduction in badger density, such as was observed in the proactively culled areas [38], would be consistent with a reduced risk of re-introduction of infection by badgers into cattle herds. However, re-infection by badgers is only one possible source of re-introduction of infection into a herd, with cattle movements being the other main potential source.

Some work has been conducted exploring the relative impacts of cattle movements and localised sources on between-herd transmission of bTB [42], however the relative contributions of cattle-to-cattle and badger-to-cattle transmission have not yet been accurately quantified, though work has been done towards modelling these interactions [43]. Certainly if cattle-to-cattle transmission was responsible for a larger degree of cattle infection than badger-to-cattle transmission, then this would be consistent with the observation that proactive culling reduces the risk of recurrence by a relatively small degree in the first instance, before the beneficial effects tail off.

The optimal control policies directed at an individual farm to reduce breakdown prolongation and recurrence, for which an individual farmer will have a vested interest, might be quite different to the optimal policies aimed at reducing incidence across a wider area. Since it is likely that future culling would be at the farmer’s expense (the Government wildlife unit that performed the culling during the RBCT has since been disbanded), the financial cost, as well as the time that farmers would have to outlay for culling operations, should be weighed against any potential beneficial/detrimental effects of the culling.

This work could be extended to examine the spatial relationships of whether localised (reactive) culling leads to prolongation or recurrence on the farm itself, and/or on contiguous farms. This might give further insight for policymakers regarding the spatial scale of effects of localised culling on persistence, and may shed more light on potential biological mechanisms regarding the interaction between badger culling and persistence.

### Conclusions

Our results suggest that a future culling policy that mirrored the proactive strategy used in the RBCT may have a marginal effect on reducing the degree of recurrence in the short-term, but this benefit is unlikely to extend much further beyond the end of the culling period. In contrast, a reactive strategy, such as that used in the RBCT, would most likely increase the average duration of breakdowns in the short-term, with little impact on reducing recurrence. These detrimental effects are unlikely to last in the long-term. In order to have any beneficial impact on recurrence, albeit most likely marginal, any culling strategy would have to mirror more closely the proactive treatment. These findings should be considered alongside those from other studies if badger culling is to form part of the future bTB control programme for cattle in GB.

## Supporting Information

Table S1 **Parameter estimates and 95% credible intervals from Bayesian model fits.**
(DOCX)

Table S2 **Counts of breakdowns in each persistence category, starting in each year and stratified by treatment group.**
(DOCX)
