# Supplementary material for: The Effect of Badger Culling on Breakdown Prolongation and Recurrence of Bovine Tuberculosis in Cattle Herds in Great Britain
Source: PLoS One. 2012 Dec 7;7(12):e51342. doi: 10.1371/journal.pone.0051342 (PMC3517421; doi:10.1371/journal.pone.0051342)
Supplement: Table S1 — Parameter estimates and 95% credible intervals from Bayesian model fits. (DOCX) [file pone.0051342.s001.docx]

**Table S1:** Parameter estimates and 95% credible intervals from Bayesian model fits.

|  |  | **Mean** | **2.5%** | **97.5%** |
| --- | --- | --- | --- | --- |
| Prolonged | Intercept | -4.43 | -13.11 | -2.17 |
|  | Confirmed (relative to unconfirmed) | 2.24 | 2.07 | 2.42 |
|  | $T_{i}^{R}$ | 0.48 | 0.07 | 0.89 |
|  | $T_{i}^{P}$ | 0.10 | -0.14 | 0.36 |
|  | $B_{i}$ | -0.06 | -0.27 | 0.14 |
|  | $B_{i}$ × $T_{i}^{R}$ | -0.30 | -0.96 | 0.35 |
|  | $B_{i}$ × $T_{i}^{P}$ | 0.14 | -0.22 | 0.52 |
|  | $t_{i}$ × $T_{i}^{R}$ | -0.51 | -0.9 | -0.12 |
|  | $t_{i}$ × $T_{i}^{P}$ | -0.13 | -0.39 | 0.14 |
|  | $t_{i}$ × Buffer × $T_{i}^{R}$ | 0.50 | -0.16 | 1.19 |
|  | $t_{i}$ × Buffer × $T_{i}^{P}$ | -0.09 | -0.48 | 0.31 |
| Recurrent  (12 months) | Intercept | -1.53 | -2.09 | -1.03 |
|  | Breakdown in past 3 years | 0.41 | 0.27 | 0.55 |
|  | Log max. herd size | 0.04 | -0.03 | 0.11 |
|  | Log total no. of reactors | 0.12 | 0.08 | 0.15 |
|  | $T_{i}^{R}$ | 0.07 | -0.32 | 0.47 |
|  | $T_{i}^{P}$ | -0.16 | -0.42 | 0.11 |
|  | $B_{i}$ | -0.13 | -0.34 | 0.09 |
|  | $B_{i}$ × $T_{i}^{R}$ | 0.17 | -0.45 | 0.77 |
|  | $B_{i}$ × $T_{i}^{P}$ | -0.01 | -0.41 | 0.40 |
|  | $t_{i}$ × $T_{i}^{R}$ | -0.17 | -0.57 | 0.25 |
|  | $t_{i}$ × $T_{i}^{P}$ | -0.01 | -0.30 | 0.28 |
|  | $t_{i}$ × Buffer × $T_{i}^{R}$ | 0.14 | -0.50 | 0.75 |
|  | $t_{i}$ × Buffer × $T_{i}^{P}$ | 0.05 | -0.4 | 0.53 |
| Recurrent  (24 months) | Intercept | -0.64 | -1.17 | -0.13 |
|  | Breakdown in past 3 years | 0.34 | 0.19 | 0.47 |
|  | Log max. herd size | 0.00 | -0.07 | 0.07 |
|  | Log total no. of reactors | 0.12 | 0.09 | 0.16 |
|  | $T_{i}^{R}$ | 0.04 | -0.28 | 0.38 |
|  | $T_{i}^{P}$ | -0.21 | -0.45 | 0.03 |
|  | $B_{i}$ | 0.02 | -0.18 | 0.22 |
|  | $B_{i}$ × $T_{i}^{R}$ | -0.22 | -0.79 | 0.35 |
|  | $B_{i}$ × $T_{i}^{P}$ | 0.08 | -0.29 | 0.44 |
|  | $t_{i}$ × $T_{i}^{R}$ | -0.01 | -0.37 | 0.33 |
|  | $t_{i}$ × $T_{i}^{P}$ | 0.19 | -0.08 | 0.48 |
|  | $t_{i}$ × Buffer × $T_{i}^{R}$ | 0.27 | -0.33 | 0.84 |
|  | $t_{i}$ × Buffer × $T_{i}^{P}$ | -0.11 | -0.54 | 0.30 |
| Recurrent  (36 months) | Intercept | -0.36 | -0.82 | 0.15 |
|  | Breakdown in past 3 years | 0.41 | 0.25 | 0.56 |
|  | Log max. herd size | 0.03 | -0.04 | 0.1 |
|  | Log total no. of reactors | 0.13 | 0.09 | 0.16 |
|  | $T_{i}^{R}$ | -0.02 | -0.35 | 0.34 |
|  | $T_{i}^{P}$ | -0.38 | -0.62 | -0.15 |
|  | $B_{i}$ | -0.12 | -0.33 | 0.11 |
|  | $B_{i}$ × $T_{i}^{R}$ | -0.26 | -0.8 | 0.28 |
|  | $B_{i}$ × $T_{i}^{P}$ | 0.38 | -0.02 | 0.74 |
|  | $t_{i}$ × $T_{i}^{R}$ | 0.13 | -0.26 | 0.49 |
|  | $t_{i}$ × $T_{i}^{P}$ | 0.52 | 0.19 | 0.84 |
|  | $t_{i}$ × Buffer × $T_{i}^{R}$ | 0.41 | -0.19 | 1.01 |
|  | $t_{i}$ × Buffer × $T_{i}^{P}$ | -0.48 | -0.94 | 0.02 |
|  |  |  |  |  |
