# Supplementary material for: The Effect of Badger Culling on Breakdown Prolongation and Recurrence of Bovine Tuberculosis in Cattle Herds in Great Britain
Source: PLoS One. 2012 Dec 7;7(12):e51342. doi: 10.1371/journal.pone.0051342 (PMC3517421; doi:10.1371/journal.pone.0051342)
Supplement: Table S2 — Counts of breakdowns in each persistence category, starting in each year and stratified by treatment group. (DOCX) [file pone.0051342.s002.docx]

**Table S2:** Counts of breakdowns in each persistence category, starting in each year and stratified by treatment group.

|  |  | **Survey** | | | **Reactive** | | | **Proactive** | | |
| --- | --- | --- | --- | --- | --- | --- | --- | --- | --- | --- |
|  | **Year** | **Controls** | **Cases** | **Proportion** | **Controls** | **Cases** | **Proportion** | **Controls** | **Cases** | **Proportion** |
| Prolonged | 1999 | 61 | 10 | 0.14 |  |  |  | 10 | 1 | 0.09 |
|  | 2000 | 103 | 22 | 0.18 | 4 | 4 | 0.5 | 24 | 4 | 0.14 |
|  | 2001 | 36 | 31 | 0.46 | 2 | 6 | 0.75 | 19 | 13 | 0.41 |
|  | 2002 | 76 | 40 | 0.34 | 24 | 13 | 0.35 | 47 | 21 | 0.31 |
|  | 2003 | 112 | 76 | 0.4 | 68 | 32 | 0.32 | 94 | 49 | 0.34 |
|  | 2004 | 109 | 56 | 0.34 | 82 | 32 | 0.28 | 84 | 45 | 0.35 |
|  | 2005 | 100 | 43 | 0.3 | 102 | 44 | 0.3 | 98 | 43 | 0.3 |
|  | 2006 | 99 | 68 | 0.41 | 99 | 40 | 0.29 | 87 | 35 | 0.29 |
|  | 2007 | 102 | 58 | 0.36 | 71 | 29 | 0.29 | 85 | 30 | 0.26 |
|  | 2008 | 119 | 53 | 0.31 | 88 | 51 | 0.37 | 101 | 32 | 0.24 |
|  | 2009 | 116 | 69 | 0.37 | 78 | 57 | 0.42 | 81 | 64 | 0.44 |
|  | 2010 | 104 | 69 | 0.4 | 77 | 51 | 0.4 | 100 | 57 | 0.36 |
|  | 2011 | 77 | 37 | 0.32 | 55 | 42 | 0.43 | 63 | 25 | 0.28 |
| Recurrent  (12 months) | 1999 | 39 | 20 | 0.34 |  |  |  | 7 | 7 | 0.5 |
|  | 2000 | 41 | 20 | 0.33 | 1 | 1 | 0.5 | 7 | 3 | 0.3 |
|  | 2001 | 30 | 9 | 0.23 | 2 | 2 | 0.5 | 14 | 9 | 0.39 |
|  | 2002 | 72 | 25 | 0.26 | 15 | 13 | 0.46 | 48 | 12 | 0.2 |
|  | 2003 | 95 | 55 | 0.37 | 53 | 30 | 0.36 | 77 | 34 | 0.31 |
|  | 2004 | 83 | 47 | 0.36 | 62 | 43 | 0.41 | 63 | 42 | 0.4 |
|  | 2005 | 72 | 43 | 0.37 | 82 | 45 | 0.35 | 85 | 32 | 0.27 |
|  | 2006 | 91 | 52 | 0.36 | 95 | 38 | 0.29 | 69 | 28 | 0.29 |
|  | 2007 | 82 | 57 | 0.41 | 50 | 49 | 0.49 | 60 | 41 | 0.41 |
|  | 2008 | 94 | 60 | 0.39 | 89 | 42 | 0.32 | 74 | 45 | 0.38 |
|  | 2009 | 121 | 47 | 0.28 | 99 | 43 | 0.3 | 90 | 36 | 0.29 |
|  | 2010 | 80 | 44 | 0.35 | 68 | 33 | 0.33 | 82 | 27 | 0.25 |
|  |  |  |  |  |  |  |  |  |  |  |
| Recurrent  (24 months) | 1999 | 38 | 21 | 0.36 |  |  |  | 7 | 7 | 0.5 |
|  | 2000 | 58 | 51 | 0.47 | 1 | 1 | 0.5 | 14 | 12 | 0.46 |
|  | 2001 | 36 | 19 | 0.35 | 2 | 4 | 0.67 | 12 | 16 | 0.57 |
|  | 2002 | 56 | 53 | 0.49 | 15 | 19 | 0.56 | 38 | 27 | 0.42 |
|  | 2003 | 81 | 90 | 0.53 | 51 | 50 | 0.5 | 67 | 55 | 0.45 |
|  | 2004 | 78 | 74 | 0.49 | 50 | 69 | 0.58 | 63 | 56 | 0.47 |
|  | 2005 | 65 | 63 | 0.49 | 74 | 76 | 0.51 | 82 | 54 | 0.4 |
|  | 2006 | 73 | 85 | 0.54 | 70 | 78 | 0.53 | 59 | 55 | 0.48 |
|  | 2007 | 66 | 86 | 0.57 | 41 | 70 | 0.63 | 52 | 58 | 0.53 |
|  | 2008 | 74 | 86 | 0.54 | 71 | 75 | 0.51 | 54 | 71 | 0.57 |
|  | 2009 | 72 | 70 | 0.49 | 67 | 56 | 0.46 | 65 | 54 | 0.45 |
| Recurrent  (36 months) | 1999 | 30 | 33 | 0.52 |  |  |  | 5 | 4 | 0.44 |
|  | 2000 | 50 | 70 | 0.58 | 1 | 2 | 0.67 | 12 | 16 | 0.57 |
|  | 2001 | 27 | 30 | 0.53 | 2 | 4 | 0.67 | 13 | 18 | 0.58 |
|  | 2002 | 41 | 69 | 0.63 | 9 | 25 | 0.74 | 32 | 33 | 0.51 |
|  | 2003 | 73 | 104 | 0.59 | 46 | 58 | 0.56 | 60 | 69 | 0.53 |
|  | 2004 | 68 | 89 | 0.57 | 44 | 78 | 0.64 | 60 | 61 | 0.5 |
|  | 2005 | 49 | 87 | 0.64 | 57 | 100 | 0.64 | 63 | 76 | 0.55 |
|  | 2006 | 48 | 112 | 0.7 | 55 | 96 | 0.64 | 45 | 70 | 0.61 |
|  | 2007 | 51 | 103 | 0.67 | 29 | 82 | 0.74 | 37 | 73 | 0.66 |
|  | 2008 | 40 | 71 | 0.64 | 40 | 79 | 0.66 | 33 | 55 | 0.62 |
